# ID-YOLOv7: an efficient method for insulator defect detection in power distribution network

**DOI:** 10.3389/fnbot.2023.1331427

**Published:** 2024-01-15

**Authors:** Bojian Chen, Weihao Zhang, Wenbin Wu, Yiran Li, Zhuolei Chen, Chenglong Li

**Affiliations:** ^1^State Grid Fujian Electric Power Research Institute, Fuzhou, China; ^2^State Grid Fujian Electric Power Co., Ltd., Fuzhou, China; ^3^College of Air Traffic Management, Civil Aviation Flight University of China, Guanghan, China

**Keywords:** insulator, defect detection, attention mechanism, YOLOv7, deep learning

## Abstract

Insulators play a pivotal role in the reliability of power distribution networks, necessitating precise defect detection. However, compared with aerial insulator images of transmission network, insulator images of power distribution network contain more complex backgrounds and subtle insulator defects, it leads to high false detection rates and omission rates in current mainstream detection algorithms. In response, this study presents ID-YOLOv7, a tailored convolutional neural network. First, we design a novel Edge Detailed Shape Data Augmentation (EDSDA) method to enhance the model's sensitivity to insulator's edge shapes. Meanwhile, a Cross-Channel and Spatial Multi-Scale Attention (CCSMA) module is proposed, which can interactively model across different channels and spatial domains, to augment the network's attention to high-level insulator defect features. Second, we design a Re-BiC module to fuse multi-scale contextual features and reconstruct the Neck component, alleviating the issue of critical feature loss during inter-feature layer interaction in traditional FPN structures. Finally, we utilize the MPDIoU function to calculate the model's localization loss, effectively reducing redundant computational costs. We perform comprehensive experiments using the Su22kV_broken and PASCAL VOC 2007 datasets to validate our algorithm's effectiveness. On the Su22kV_broken dataset, our approach attains an 85.7% mAP on a single NVIDIA RTX 2080ti graphics card, marking a 7.2% increase over the original YOLOv7. On the PASCAL VOC 2007 dataset, we achieve an impressive 90.3% mAP at a processing speed of 53 FPS, showing a 2.9% improvement compared to the original YOLOv7.

## 1 Introduction

With the growing demand for power, the power distribution network, as a critical component of power systems, plays a pivotal role in transmitting electricity from power stations to end-users. Within the power distribution network, insulators are essential components used extensively to ensure the safe and stable operation of the electrical system (Lei and Sui, 2019). However, insulators in power distribution networks may suffer from defects such as self-detonation, corrosion, and breakage due to long-term climate changes, pollution, and mechanical vibration. According to relevant data, accidents caused by insulator defects rank at the forefront in power systems (Kim et al., 2019; Stefenon et al., 2019; Zheng et al., 2022). Hence, it is essential to conduct regular inspections of insulators within the power distribution network, promptly detecting defective insulators and performing necessary maintenance.

In the past, traditional methods for detecting insulator defects primarily relied on manual inspections. This way is not only time-consuming and laborious but also often inadequate for covering all insulators within large-scale power distribution network. In addition, manual inspections are susceptible to human factors, which can significantly increase the risk of false and omission detection. With the development of technology, non-contact detection technologies such as infrared (Zheng H. et al., 2020), ultraviolet (Liu et al., 2021), ultrasonic (Tian et al., 2019), and so on are used in the field of electric power inspection, so as to fulfill the defect detection of insulators. However, the high cost of this equipment and the susceptibility to background interference are significant limitations in practical applications.

In recent years, due to the rapid developments in unmanned aerial vehicle (UAV) inspection technology and computer vision techniques, traditional methods of manual inspection are gradually being replaced. Traditional visual detection methods primarily involve the extraction of features such as contours, colors, and textures of insulators. These features are then combined with machine learning techniques like Support Vector Machines and Cluster Analysis to achieve insulator state detection in images (Zhai et al., 2018; Fang et al., 2020; Tan et al., 2020). However, traditional methods often struggle to handle diverse and complex insulator defect scenarios. They can be limited in capturing the variety of insulator defects effectively and are susceptible to factors like noise and background interference, resulting in low accuracy and limited generalization.

In contrast, deep learning-based object detection techniques are opening up new possibilities for insulator defect detection. Deep learning architectures leverage Convolutional Neural Networks (CNNs) to automatically learn deep features layer by layer from images. They optimize network model parameters through training on large-scale data, thereby enhancing detection accuracy. Currently, deep learning has demonstrated remarkable achievements across various domains, such as autonomous driving (Nguyen et al., 2018), medical diagnostics (Bakator and Radosav, 2018), and computer vision (Voulodimos et al., 2018). At the same time, experts have gradually shifted their focus to the field of electrical equipment inspection, especially in the insulator defect detection (Prates et al., 2019; Niu et al., 2023). Deep learning, with its remarkable generalization and cross-scenario adaptability, is ushering in a revolutionary transformation in insulator defect detection. This transformation not only markedly improves the efficiency and accuracy of the detection process, but also opens up entirely new prospects for the reliability and safety of the power systems. However, deep learning-based insulator defect detection methods still face several challenges when dealing with large field-of-view and multi-angle images captured by drones. In particular, complex background environments and subtle defect objects in the images can interfere with the accuracy and reliability of defect detection. Therefore, further research and solutions are needed to address these issues.

To address the high false and omission detection rates in insulator defect detection in power distribution network, we propose the ID-YOLOv7 model based on the YOLOv7 algorithm. Firstly, we make a detailed analysis for the captured insulator images, and propose an Edge Detail Shape Data Augmentation (EDSDA) method. This method expands the training dataset, enhances the model's robustness, and guides the model to pay more attention to insulator shape information. Secondly, in order to enhance the model's capacity for capturing features from subtle insulator defects and to tackle the problem of feature loss in the deep networks of YOLOv7 due to reduced image channels, we draw inspiration from the EMA (Ouyang et al., 2023) module and design a Cross Channel and Spatial Multi-scale Attention (CCSMA) module. This module is capable of integrating contextual information from different scales within the network, allowing the model to achieve better pixel-level focus on higher-level feature maps. Subsequently, we introduce the Bi-directional Concatenation module to reconstruct the Neck component of the network. This innovative structural design ensures adequate information transfer between feature layers and avoids loss of important features. Finally, the MPDIoU (Siliang and Yong, 2023) loss function is used to calculate the localization loss, which improves the convergence speed of the model and reduces redundant computational cost.

The main contributions of this paper are as follows:

- In this study, we design the ID-YOLOv7 algorithm based on YOLOv7. We restructure the Neck component and create a Re-BiC module for multi-scale feature fusion. This enhancement addresses the issue of feature information loss during inter-feature layer interaction. Additionally, during model training, we employ the MPDIoU function to calculate the localization loss, thereby expediting the model's convergence rate and reducing redundant computational costs. Our model exhibits significant advantages in insulator defect detection tasks.
- We propose an Edge Detail Shape Data Augmentation (EDSDA) method that expands the training set while increasing the model's sensitivity to insulator's edge shape. Meanwhile, We create a Cross Channel and Spatial Multi-scale Attention (CCSMA) Module to strengthen the network's attention to high-level feature maps, which increases the detection accuracy of subtle defects of insulators.
- We conduct a comprehensive series of experiments to validate the efficacy of our approach. The experimental results affirm that our method attains state-of-the-art performance on the Su22kV_broken and PASCAL VOC 2007 datasets. Specifically, the ID-YOLOv7 algorithm achieves 85.7% mAP on the Su22kV_broken dataset and 90.3% mAP on the PASCAL VOC 2007 dataset at a speed of 53 FPS.

The remainder of this article is structured as follows. Section 2 provides a comprehensive review of related work in the field of insulator defect detection. Section 3 provides a detailed description of the ID-YOLOv7 algorithm and the insulator defect dataset. In Section 4, we presents the results of ablation experiments conducted on our proposed algorithm, as well as a performance comparison with other state-of-the-art algorithms on the Su22kV_broken datasets and the PASCAL VOC 2007 datasets. Finally, Section 5 summarizes the article and discusses our future research directions.

## 2 Related work

### 2.1 Conventional methods for insulator defect detection

To identify insulator defects in UAV inspection photos, conventional insulator defect detection methods apply diverse techniques such as contour detection, color feature analysis, and shape-texture feature analysis. Tan et al. (2020) present a fusion technique for detecting catenary insulators based on shed shape characteristics and gray similarity matching. Zhai et al. (2018) identify the target region of the insulator based on color and spatial features, and morphologically processes the target region to detect the fault location of the insulator. Yu et al. (2019) offer an active contour model that takes into account insulator texture and shape information. They devise a novel convex energy function, leveraging texture features extracted from a semi-local region descriptor. However, this method requires the acquisition of a priori knowledge of shape and has low applicability. Fang et al. (2020) introduce color and distance factors, optimizing the algorithm by integrating a priori information, this enhancement enables the algorithm to effectively avoid insulator false negative and false positive.

### 2.2 Deep learning based insulator defect detection

With the continuous development of deep learning, numerous methods related to insulator defect detection are emerging. The research in this field can be broadly categorized into two main groups. The first category comprises two-stage object detection models, exemplified by R-CNN (Girshick et al., 2014), Faster R-CNN (Ren et al., 2015), and Mask R-CNN (He et al., 2017). Two-stage object detection models achieve improved detection accuracy by training region proposal networks to generate candidate boxes, and subsequently performing classification and regression operations on these candidate regions. Zheng R. et al. (2020) use an R-CNN-based CNN approach to extract visual features from inspection images and detect insulator self-explosion defects. This approach can identify insulator and defect sites under a variety of environmental circumstances. Liao et al. (2019) propose a Faster R-CNN technique in combination with a deep residual network, ResNet101. Soft Non-Maximum Suppression (Soft-NMS) is also used to improve the identification of overlapping insulators. However, this algorithm involves a substantial computational load and does not meet the real-time requirements for insulator defect detection. Wen et al. (2021) propose two Faster R-CNN-based approaches: Exact R-CNN and CME-NN. In CME-NN, they employ an encoder-decoder mask extraction network to mitigate the influence of complex environments and subsequently employ Exact R-CNN to detect the defective insulator locations. Tan et al. (2022) improve Mask R-CNN by gradient, texture, and gray feature fusion (GTGFF) along with K-mean clustering analysis model for insulator detection in high-speed railways. However, this method is limited by the relatively uniform types of insulators.

Another category comprises one-stage object detection models represent by the You Only Look Once(YOLO) series (Redmon et al., 2016; Redmon and Farhadi, 2017, 2018; Bochkovskiy et al., 2020; Jocher, 2020; Wang C.-Y. et al., 2023) and the Single Shot MultiBox Detector (SSD) algorithm (Liu et al., 2016). The one-stage method does not require region proposal networks, and the input data is directly classified and regressed after the training of the backbone feature extraction network, which can effectively shorten the training and inference time. Han et al. (2023) introduce the DSMH-YOLOv4 algorithm for insulator defect detection. Building upon YOLOv4, they improve the residual structures and CSPDarknet53 backbone model and incorporate the SA-Net attention model. This not only reduces the model's parameter count but also enhances attention to target features. Xu et al. (2022) propose an improved yolov4, introducing the lightweight module Mobilet-V1 and the spatial and channel squeeze and channel excitation attention mechanism module, and the depthwise separable convolution is used to reduce the network parameters. Guo et al. (2023) propose an improved insulator detection algorithm based on YOLOv5, which combines a segmentation head network utilizing self-attention and transformer (HST-Net) to identify and assess the extent and type of damage on insulator surfaces. Hu et al. (2023) introduce the BiFPN module into the YOLOv5s network for feature fusion. They incorporate the SPD module to enhance the extraction of features related to small objects and introduce the CBAM attention mechanism to augment the model's focus on insulator defect regions, thereby improving detection accuracy. Miao et al. (2019) propose a method for automatically extracting multi-level characteristics from images that combines the SSD model with a two-stage fine-tuning procedure. This approach allows for the rapid and accurate detection of porcelain and composite insulators in complicated backgrounds.

Compared to the aforementioned methods, the YOLOv7 model proposed by Wang C.-Y. et al. (2023) has higher characterization capabilities, presenting faster and more accurate performance on the COCO dataset. YOLOv7's architecture contains several bag-of-freebies strategies targeted at improving object detection accuracy without raising the inference load. Additionally, it utilizes a re-parameterized model to replace the original modules, effectively handling different layer outputs through dynamic label assignments from coarse to fine-grained levels. This algorithm not only supports mobile GPUs and GPU devices from the edge to the cloud, but also excels in speed and accuracy in a range from 5 to 120 FPS. However, the current research on the application of the YOLOv7-based model in the field of insulator defect detection is still insufficient. Meanwhile, there is still room for improving the accuracy of the model in insulator defect detection (Zheng et al., 2022). In this context, addressing the specific challenges posed by complex backgrounds and small defective targets in power distribution networks, this paper presents significant enhancements to the original YOLOv7 framework. By introducing novel techniques and strategies into the model, the objective is to achieve higher defect detection accuracy and greater robustness, thereby providing more effective support for the reliable operation of power distribution systems.

The conclusions drawn from the above research indicate that defect detection methods for insulators are often constrained by complex backgrounds and small defects, thereby necessitating further improvement in detection accuracy. Here, we provide a summary of these methods:

- Conventional insulator defect detection algorithms can quickly compute and achieve satisfactory detection results when dealing with images with simple backgrounds and distinct features. However, these approaches rely substantially on the feature extractor's integrity, need high-quality input images, and are subject to glare and background interference.
- Deep learning based algorithms for insulator defect detection exhibit outstanding performance. However, two-stage and one-stage object detection algorithms each possess distinct advantages. The former has a complex structure, higher detection accuracy, and relatively slower speed, while the latter has a simple structure, rapid detection speed, but relatively lower accuracy.
- The images of power distribution network insulators obtained through UAV inspections encompass diverse background elements. Simultaneously, insulator defects may occupy only a small portion of the entire image, resulting in very small defect targets. These factors can impact detection performance. As a result, there is still potential for improvement in improving the accuracy of insulator defect detection.

## 3 Materials and methods

### 3.1 Dataset preparation and analysis

**Figure 1:**
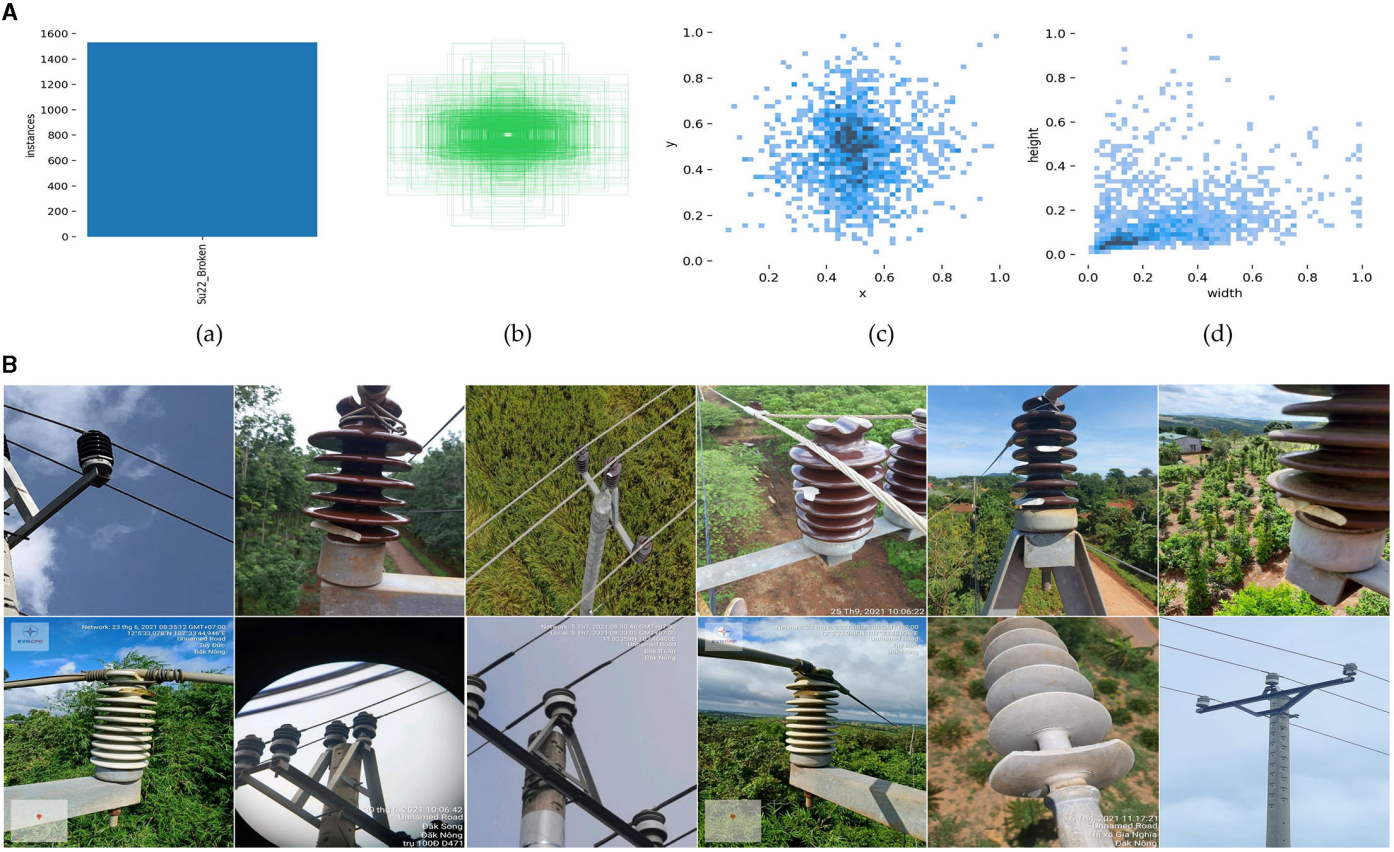
**(A)** Label distribution:(a) number of labels; (b) visualization of label box; (c) label position; (d) label size. **(B)** Image samples of the Su22kV_broken dataset.

In this paper, we use the Su22kV_broken dataset, which is provided by a private user of the Roboflow platform. It encompasses a total of 1,236 images, each with a resolution of 512 × 512. Since this dataset aims at detecting defective conditions that exist in insulators of the power distribution network, this dataset is only labeled with insulator defective parts. At the same time, omissions and errors in the labeling results were corrected. We divide the modified dataset into training set, validation set and test set in the ratio of 8:1:1. We count the number and distribution of tags in the dataset and the results are shown in Figure 1A.

As shown in Figure 1A, (a) depicts the total number of labels Su22kV_Broken, indicating that there are enough insulator defect examples in the dataset to allow the training and learning of subsequent deep learning models. (b) depicts the label box distribution in the dataset, whereas in (c), the horizontal coordinate represents the ratio of the label center's horizontal coordinate to the image width, and the vertical coordinate represents the ratio of the label center's horizontal coordinate to the image height. Labels are uniformly distributed throughout the dataset and tend to be centered in the middle of the image, as seen in (b) and (c). The width of the horizontal coordinate in (d) represents the ratio of the label width to the image width, and the height of the vertical coordinate represents the ratio of the label height to the image height. The dataset exhibits a higher frequency of small objects.

In addition, Figure 1B shows some image data samples of Su22kV_broken dataset, from which it can be seen that the background of power distribution network insulator images is very complex. At the same time, influenced by the image acquisition angles and distances, many insulators exhibit subtle defects. This complexity brings more challenges for the whole object detection task.

### 3.2 Proposed method

**Figure 2:**
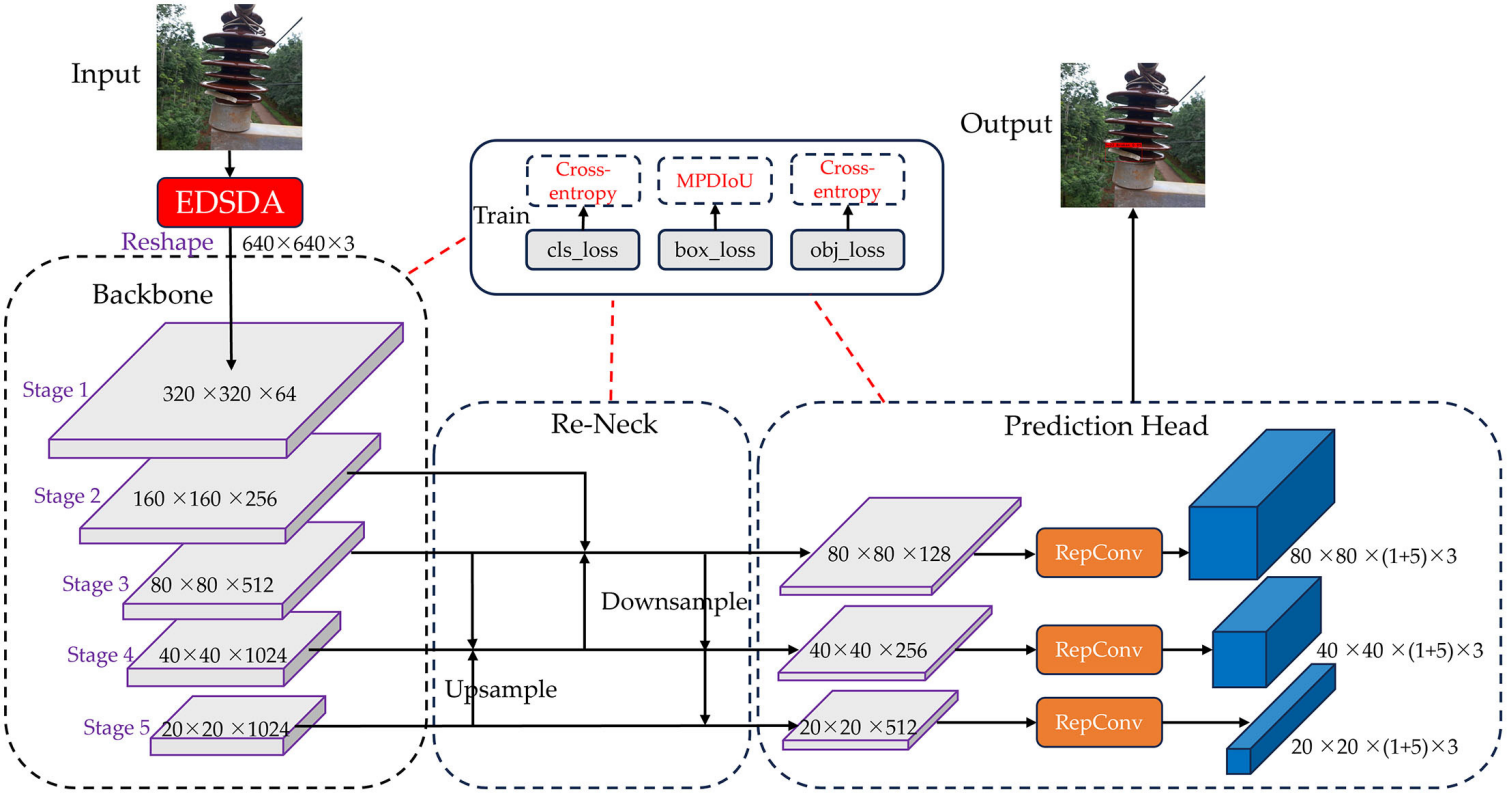
The general architecture of our proposed ID-YOLOv7 network. In the Re-Neck component, we continue to employ the Feature Pyramid Network (FPN) approach. However, a key distinction lies in the upsampling process, which effectively involves the iterative fusion of adjacent three-layer features.

In the realm of one-stage object detection algorithms, YOLOv7 (Wang C.-Y. et al., 2023) has exhibited superior detection accuracy compared to YOLOv5 (Jocher, 2020), while also preserving robust real-time performance. In this study, we choose the YOLOv7 as the baseline algorithm for detecting insulators defects in power distribution network. We use YOLOv7 algorithm to train Su22kV_broken dataset, and through testing we found the original YOLOv7 algorithm has more prominent omissions and false detection when facing complex background images and subtle defects of insulators. Therefore, we propose an improved YOLOv7 method for detecting insulator defects, named ID-YOLOv7. The primary objective is to improve the accuracy of detecting insulator defects by making improvements to YOLOv7 in three key aspects: data augmentation, network structure, and loss function. The general architecture of the ID-YOLOv7 network is depicted in Figure 2.

#### 3.2.1 Edge detail shape data augmentation

In the field of deep learning, commonly data augmentation strategies include random flip, random crop, chromaticity transform, saturation transform, etc., which are designed to expand the number of training sets so as to alleviate the overfitting problem and improve the robustness of the model. The Mosaic data augmentation method is used in YOLOv4, YOLOv5 and YOLOv7, which enriches the background information of the images while expanding the training set. However, the primary focus of this study is detecting insulators defects in power distribution network. As elucidated through the analysis of the Su22kV_broken dataset in Section 3.1, insulators in power distribution network are predominantly located in rural mountainous areas. The captured images often exhibit complex backgrounds characterized by dense shrubbery and numerous trees. Moreover, insulators defects in power distribution network tend to be less conspicuous. Consequently, these factors collectively heighten the difficulty associated with detecting insulator defects.

By analyzing defective insulators, we find that the shape of insulators will change significantly after defects occur. And when we judge whether an insulator is defective or not, the first thing we usually focus on is the shape characteristics of the insulator. Hence, it is imperative to enhance the sensitivity of the neural network model on insulator shape information, enabling it to rely more heavily on insulator shapes for defect detection. This improvement will better distinguish defects.

**Figure 3:**
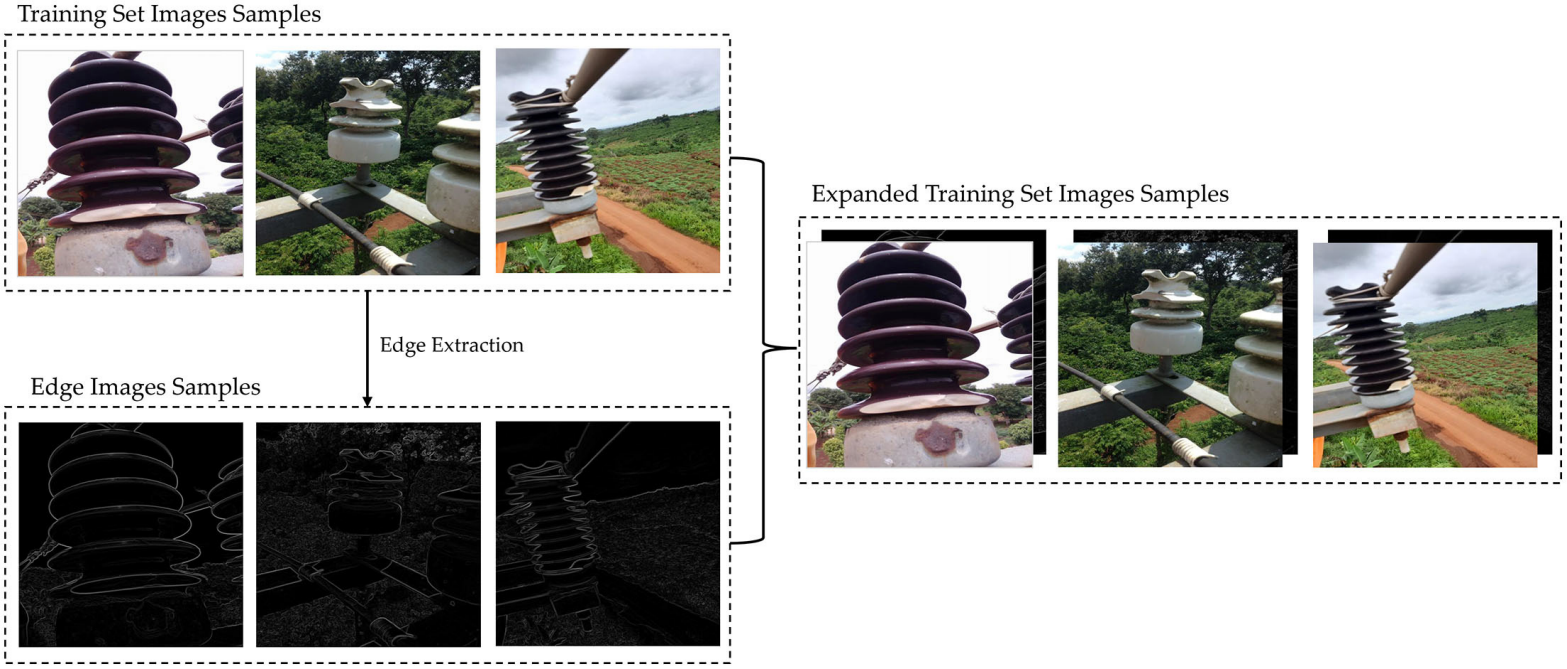
Implementation of edge detail shape data augmentation (EDSDA).

In summary, this paper proposes an augmentation method for edge detail shapes on the original YOLOv7 algorithm, aiming to enhance the neural network's focus on insulator edge detail shapes, as depicted in Figure 3. In a detailed fashion, we begin by utilizing an image edge extraction algorithm to generate edge detail images for the training set of the Su22kV_broken dataset. Considering that the YOLOv7 network requires RGB three-channel images as input, whereas the edge detail images are monochromatic, we replicate the edge detail image twice and merge them to construct a three-channel edge detail image. Following this, the expanded three-channel edge detail image is incorporated into the Su22kV_broken training set. Since the edge detail images primarily capture the shape and texture of insulators, the expanded edge detail images can guide the model to pay more attention to the shape features of the insulator during the model's training process, thereby improving the detection accuracy of insulator defect.

#### 3.2.2 Cross channel and spatial multi-scale attention module

Within the original YOLOv7 backbone architecture, the stacking of numerous convolutional blocks poses a potential challenge. This is because, as the image channels progressively decrease, it becomes more likely that the features of subtle defect of insulator within the image might be lost or compromised. Hence, to bolster the model's attention toward features of subtle defects of insulators, we incorporate an attention mechanism module within our ID-YOLOv7 network. Attention mechanisms are typically classified into three categories: channel attention, spatial attention, and channel-spatial hybrid attention mechanisms. One of the prominent models for channel attention is SENet (Hu et al., 2018), which includes two components: compression and excitation. The compression part aims to reduce global spatial information, followed by channel-wise feature learning to determine the significance of each channel. Subsequently, the excitation part allocates varying weights to individual channels. STN (Jaderberg et al., 2015) stands out as a model for spatial attention, as it can transform deformed data in spatial dimensions and automatically capture crucial region features, ensuring that the image yields the same results as the original image after undergoing operations like cropping or translation during data augmentation. The CBAM (Woo et al., 2018) model serves as an exemplary model for channel-spatial mixed attention, primarily designed for feedforward convolutional neural networks. When presented with an intermediate feature map, the CBAM module progressively generates attention maps along two distinct dimensions (channel and spatial). Subsequently, it performs adaptive feature optimization through element-wise multiplication with the input feature map.

The models mentioned above excel in producing highly distinguishable feature representations during model inference. Nevertheless, the approach of modeling cross-channel relationships through channel dimension reduction may inadvertently lead to unintended consequences in feature extraction for subtle defects of insulator. Hence, we introduce the concept of a multiscale attention module (EMA) (Ouyang et al., 2023) and improve it to propose the cross-channel and spatial multiscale attention module (CCSMA).

**Figure 4:**
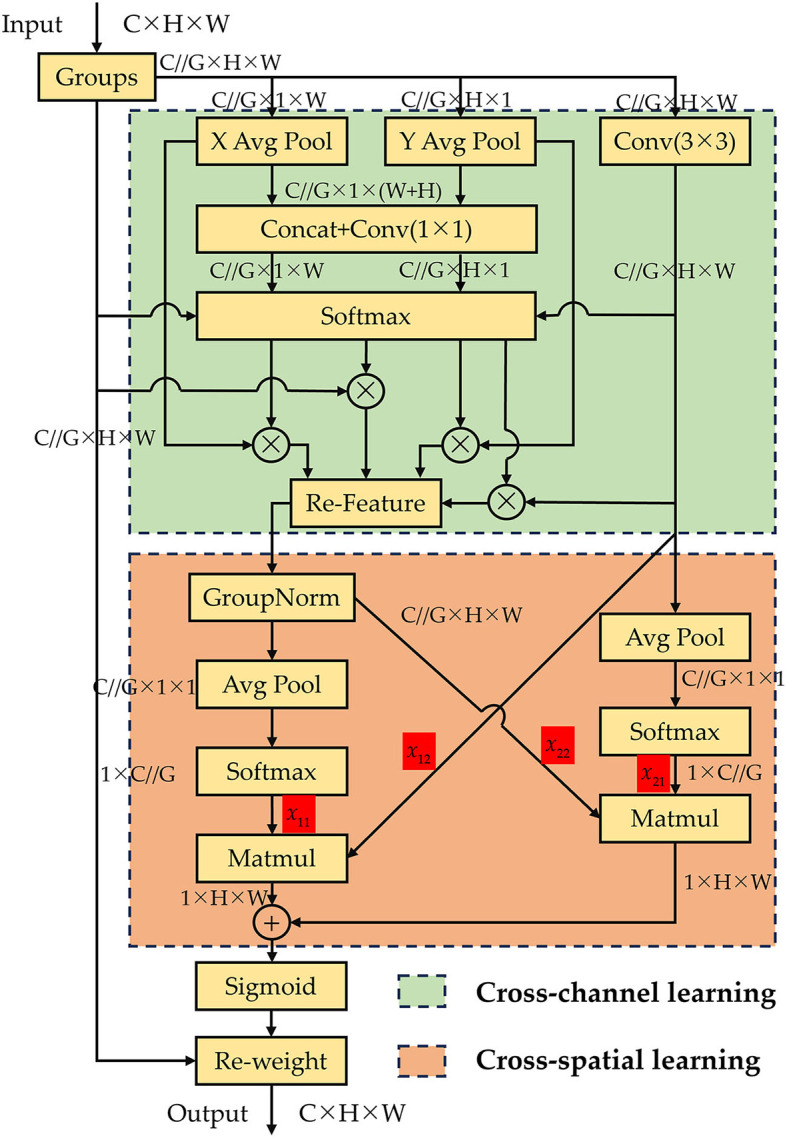
The proposed CCSMA network.

As depicted in Figure 4, the CCSMA module utilizes parallel substructures, effectively eliminating additional sequential processing in the entire network, thereby expediting the inference process. The CCSMA module is divided into two primary components: Cross-channel learning and Cross-spatial learning. It utilizes three parallel pathways to extract attention weight descriptors for grouped feature maps, comprising two parallel 1 × 1 branches and one 3 × 3 branch. Within the Cross-channel learning segment, two parallel 1 × 1 branches encode the channels by employing two global average pooling operations. Afterward, the features from these two branches are concatenated and subjected to grouped 1 × 1 convolution. These amalgamated features, in conjunction with the features from lower layers and the 3 × 3 convolution branch, are concurrently fed into a softmax function for linear transformation fitting. This process yields adaptive weight values for each branch, subsequently facilitating weighted summation for feature recombination, ultimately producing the output of the Cross-channel learning section. The output section of Cross-channel learning can be expressed using the Equations (1) and (2).


(1) $$\begin{array}{l} a_{i}=\frac{\exp\left(F_{i}\right)}{\overset{n}{\underset{i=1}{\sum }}\exp\left(F_{i}\right)} \end{array}$$

(2) $$\begin{array}{l} output_{c}=\overset{n}{\underset{i}{\sum }}F_{i}⊗a_{i} \end{array}$$

Where *F*_*i*_ denotes the feature vectors fed into the softmax function, comprising features from the Groups, X, Y, and Conv(3 × 3) layers. And *a*_*i*_ denotes the weight values computed for each vector after undergoing the Softmax function, while *output*_*c*_ denotes the features restructured by the Cross-channel learning network.

Following the modeling operation for cross-channel information interaction in the channel direction, the network attains multi-scale feature representations. This process not only involves encoding information between channels to fine-tune the importance of various channels, but also retaining accurate spatial structural information within those channels. Expanding on this foundation, the features derived from the Cross-channel learning module and those originating from the 3 × 3 output are subjected to 2D global average pooling operations, respectively. Following this, they are each input into the Cross-spatial learning module to generate two consolidated spatial attention weight sets. Ultimately, these weights are combined with features from lower layers via a sigmoid function to produce the output features. 2D global average pooling operation can be expressed using Equation (3).


(3) $$\begin{array}{l} Z_{c}=\frac{1}{H\times W}\overset{H}{\underset{j}{\sum }}\overset{W}{\underset{i}{\sum }}x_{c}\left(i,j\right) \end{array}$$

Where *H* and *W* denote the height and width dimensions of the input features, and *x*_*c*_ signifies the input feature for the channel c. And the output of the Cross-spatial learning network can be represented using Equations (4) and (5).


(4) $$\begin{array}{l} \delta =Sigmoid\left(x_{11}\cdot x_{12}+x_{21}\cdot x_{22}\right) \end{array}$$

(5) $$\begin{array}{l} Output_{s}=Groups⊗\delta \end{array}$$

Where δ denotes the weight values output after being processed by the Sigmoid function, and *Groups* denotes the grouped features input into the network. *Output*_*s*_ denotes the features ultimately output after undergoing processing by the Cross-spatial learning module.

The CCSMA module is capable of integrating contextual information from different scales within our ID-YOLOv7 network, facilitating improved pixel-level focus on higher-level feature maps, particularly for subtle defect features of insulator. Moreover, the CCSMA module ensures that the final output size matches the input size, enabling efficient integration within YOLOv7.

#### 3.2.3 Loss function

In the original YOLOv7, the loss function consists three components: confidence loss (*L*_*obj*_), classification loss (*L*_*cls*_), and localization loss (*L*_*box*_). As shown in Equation (6), the total loss function of the YOLOv7 model is the weighted sum of the three individual loss components. Specifically, the confidence loss and classification loss are both computed using the binary cross-entropy function, while the localization loss is calculated using the CIOU loss function. The confidence loss component serves the purpose of discerning whether a feature point contains an object, the classification loss component is utilized to classify the object category within the feature point, and the localization loss component is employed to determine the regression parameters of the feature point. During the training process, after positive sample matching, the model obtains the corresponding prior boxes for each genuine bounding box. All prior boxes corresponding to genuine bounding boxes are labeled as positive samples, while the remaining prior boxes are designated as negative samples. Cross-entropy loss is computed based on the predictions for positive and negative samples, coupled with whether the feature point contains an object. The resulting calculations are used as the output for the confidence loss component. Upon acquiring the corresponding prior boxes for each bounding box, the model extracts the class prediction results from these prior boxes. Cross-entropy loss is then calculated based on the true box categories and the class prediction results of the prior boxes, with the computed results serving as the output for the classification loss component. Additionally, the CIOU loss is computed using the true boxes and predicted boxes, and the resulting calculations are utilized as the output for the localization loss component.


(6) $$\begin{array}{l} Loss_{YOLOv7}=L_{obj}\times \omega_{1}+L_{cls}\times \omega_{2}+L_{box}\times \omega_{3} \end{array}$$

Where ω_1_, ω_2_, and ω_3_, respectively, represent the weight coefficients of *L*_*obj*_, *L*_*cls*_, and *L*_*box*_.

**Figure 5:**
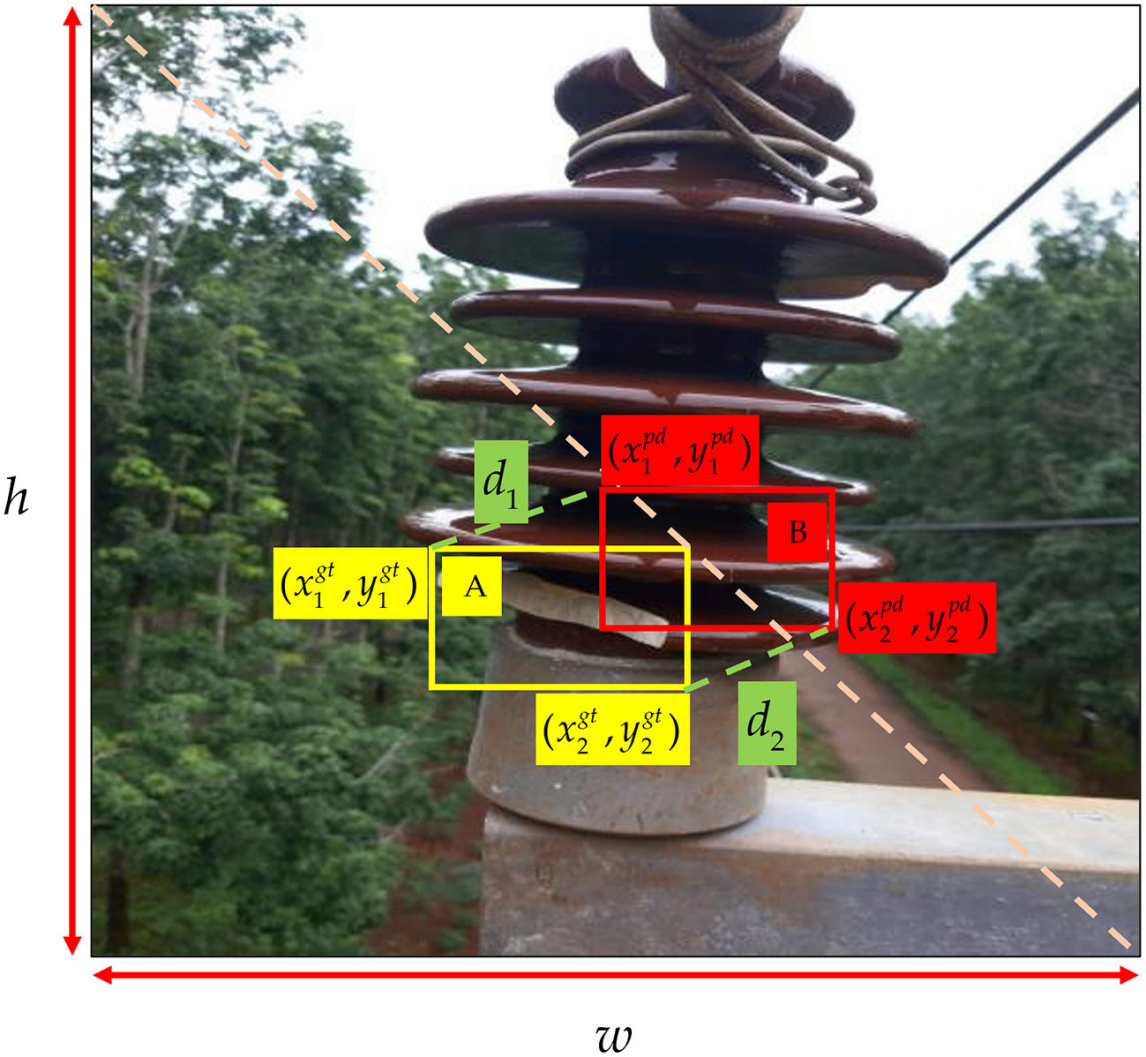
Factors of *L*_*MPDIoU*_.

To improve the training efficacy of bounding box regression, expedite convergence, and enhance regression accuracy during model training, we utilize the MPDIoU (Siliang and Yong, 2023) function to compute the localization loss component. The MPDIoU loss function presents a novel metric grounded in intersection over union (IoU), as illustrated in Figure 5. It aims to minimize the distance between the top-left and bottom-right points of the predicted bounding box and the ground truth box. The fundamental principles are elucidated in Equations (7) through (10).

The yellow box represents the ground truth box, while the red box represents the predicted box. $\left(x_{1}^{gt},y_{1}^{gt}\right)$ denotes the coordinates of the top-left point of the ground truth box, $\left(x_{2}^{gt},y_{2}^{gt}\right)$ represents the coordinates of the bottom-right point of the ground truth box, $\left(x_{2}^{pd},y_{2}^{pd}\right)$ signifies the coordinates of the top-left point of the predicted box, $\left(x_{1}^{pd},y_{1}^{pd}\right)$ represents the coordinates of the bottom-right point of the ground truth box, *d*_1_ and *d*_2_, respectively, indicate the distances between the top-left and top-left, and bottom-right and bottom-right points of the ground truth and predicted boxes. Equations (7) and (8) can be used to calculate *d*_1_ and *d*_2_:


(7) $$\begin{array}{l} d_{1}^{2}={\left(x_{1}^{pd}-x_{1}^{gt}\right)}^{2}+{\left(y_{1}^{pd}-y_{1}^{gt}\right)}^{2} \end{array}$$

(8) $$\begin{array}{l} d_{2}^{2}={\left(x_{2}^{pd}-x_{2}^{gt}\right)}^{2}+{\left(y_{2}^{pd}-y_{2}^{gt}\right)}^{2} \end{array}$$

Subsequently, the final *L*_*MPDIoU*_ can be calculated based on *d*_1_ and *d*_2_ through Equations (9) and (10).


(9) $$\begin{array}{l} MPDIoU=\frac{A\cap B}{A\cup B}-\frac{d_{1}^{2}}{w^{2}+h^{2}}-\frac{d_{2}^{2}}{w^{2}+h^{2}} \end{array}$$

(10) $$\begin{array}{l} L_{MPDIoU}=1-MPDIoU \end{array}$$

The MPDIoU loss function simplifies the similarity comparison between two bounding boxes, allowing it to accommodate both overlapping and non-overlapping bounding box regressions. Moreover, all elements of existing bounding box regression loss functions can be expressed using the four point coordinates, as illustrated in Equations (11)–(13).


(11) $$\begin{array}{l} \left|C\right|=(\max(x_{2}^{gt},x_{2}^{pd})-\min(x_{1}^{gt},x_{1}^{pd}))\times (\max(y_{2}^{gt},y_{2}^{pd})\\ -\min(y_{1}^{gt},y_{1}^{pd})) \end{array}$$

(12) $$\begin{array}{l} x_{c}^{gt}=\frac{x_{1}^{gt}+x_{2}^{gt}}{2},y_{c}^{gt}=\frac{y_{1}^{gt}+y_{2}^{gt}}{2},x_{c}^{pd}=\frac{x_{1}^{pd}+x_{2}^{pd}}{2},y_{c}^{pd}=\frac{y_{1}^{pd}+y_{2}^{pd}}{2} \end{array}$$

(13) $$\begin{array}{l} w_{gt}=\left|x_{2}^{gt}-x_{1}^{gt}\right|,h_{gt}=\left|y_{2}^{gt}-y_{1}^{gt}\right|,w_{pd}=\left|x_{2}^{pd}-x_{1}^{pd}\right|,\\ h_{pd}=\left|y_{2}^{pd}-y_{1}^{pd}\right| \end{array}$$

|*C*| represents the area of the minimum bounding rectangle of the ground truth and predicted boxes, $\left(x_{c}^{gt},y_{c}^{gt}\right)$ and $\left(x_{c}^{pd},y_{c}^{pd}\right)$, respectively, denote the center coordinates of the ground truth and predicted boxes, *w*_*gt*_ and *h*_*gt*_ represent the width and height of the ground truth box, while *w*_*pd*_ and *h*_*pd*_ signify the width and height of the predicted box. Through Equations (11)–(13), we can also calculate deviations for non-overlapping regions, center point distances, width, and height. This approach not only ensures comprehensive consideration but also streamlines the calculation process. Therefore, in the localization loss component of ID-YOLOv7 model, we opt for employing the MPDIoU function to compute the loss.

#### 3.2.4 Reconstructed neck component

**Figure 6:**
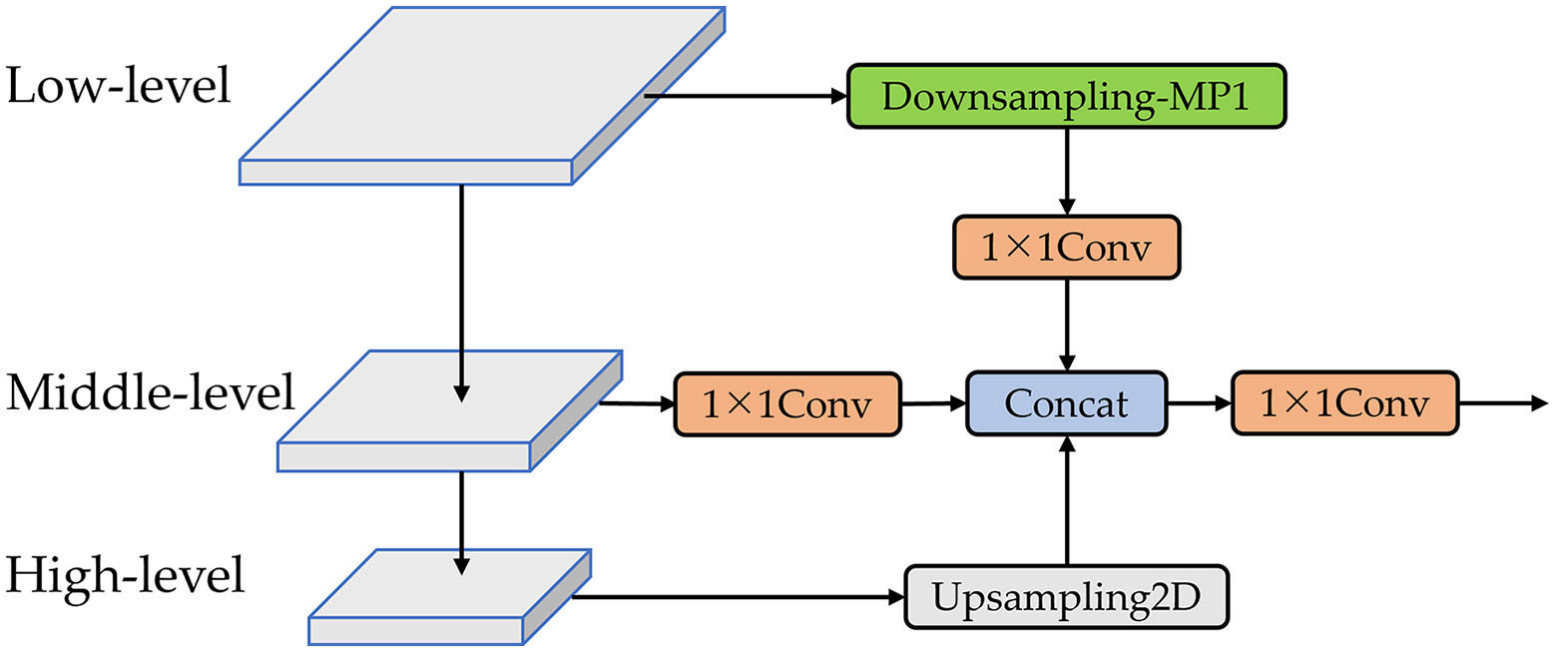
The Re-BiC network. The downsampling-MP1 module is formed by the concatenation of two branches. One branch comprises an max pooling layer followed by a CBS block, while the other branch consists of two stacked CBS blocks. A detailed illustration of the downsampling-MP1 module is provided in Figure 7.

**Figure 7:**
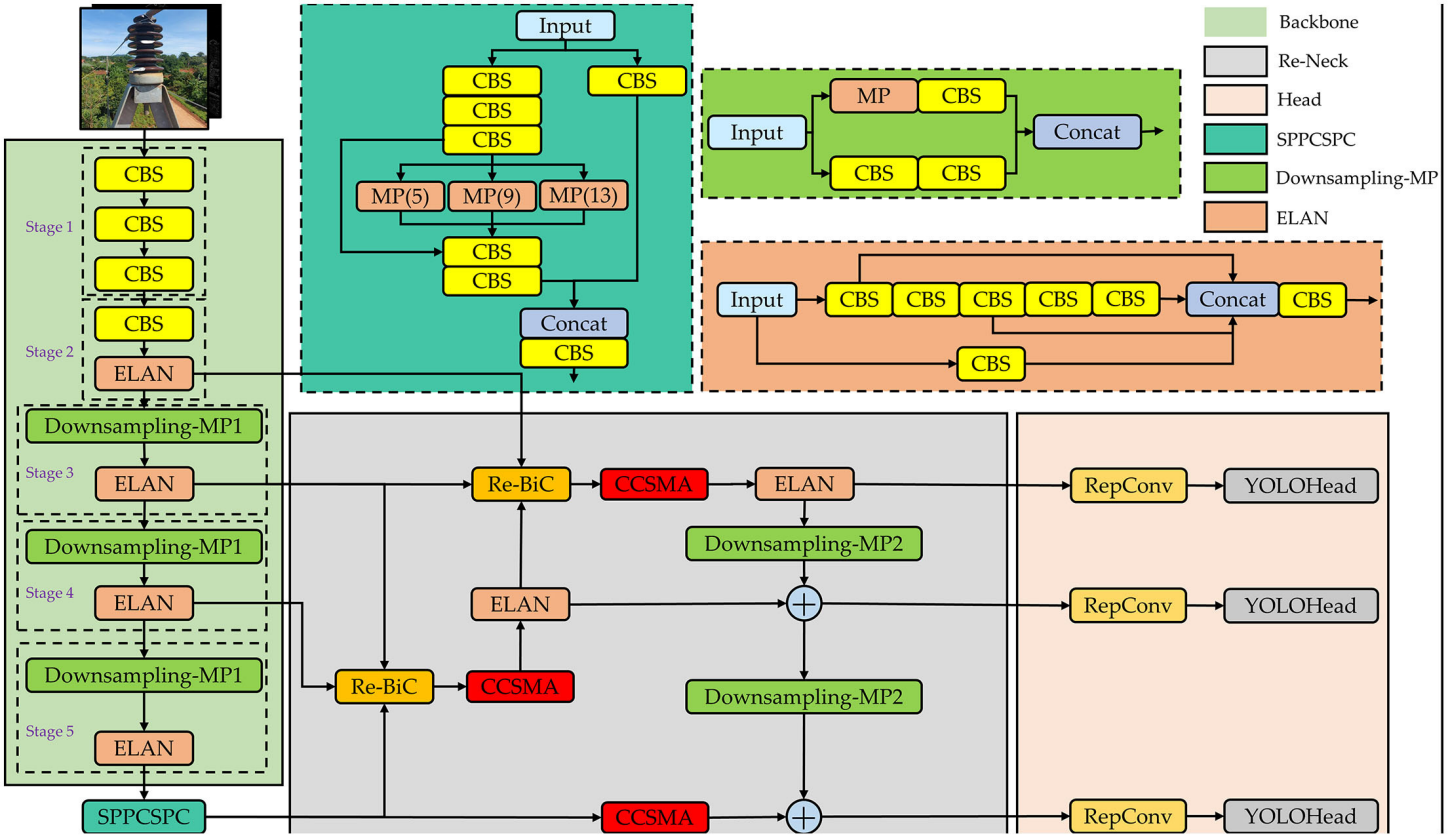
Overview of the ID-YOLOv7 network. The output channel count remains consistent with the input channel count after the feature map undergoes downsampling-MP1 processing. However, after downsampling-MP2 processing, the output channel count is adjusted to twice the input channel count.

In the original YOLOv7 architecture, the Neck component consists of Path Aggregation Network (PANet) and Feature Pyramid Network (FPN). FPN offers an efficient architectural design that enhances detection accuracy for objects of various sizes by fusing multi-scale features through cross-scale connections and information exchange (Wang C. et al., 2023). However, in the traditional FPN structure, the interaction of information between layers is acquired through a layer-by-layer recursive manner, potentially resulting in the loss of critical features when exchanging information between lower and higher layers. To address this issue, we combine the concept of Bi-directional Concatenation (Li et al., 2023) to design a Re-BiC structure for multi-scale feature fusion, as illustrated in Figure 6. Simultaneously, we reconstruct the Neck component, as depicted in Figure 7.

Utilizing the Re-BiC module for multi-scale information interaction among the high, medium, and low-level features within the Backbone, we initially pass the low-level features through a Downsampling-MP1 module and a 1 × 1 convolution for downsampling processing, resulting in the output size half of the input size and the output channel count reduced to half of the input. The middle-level features undergo a 1 × 1 convolution, maintaining their size while reducing the channel count to one-fourth of the input. The high-level features are subjected to Upsampling2D, doubling their output size while retaining the same channel count. Subsequently, these three processed feature maps are concatenated and then passed through a 1 × 1 convolution operation for dimensionality reduction.

As illustrated in Figure 7, we input image data that has undergone image augmentation into the network. It initially undergoes processing by the network's Backbone, which consists of CBS blocks, ELAN modules, and Downsampling-MP1 modules. The CBS block is composed of Convolutional, Batch Normalization, and SiLU activation layers. The ELAN module is constructed by stacking multiple CBS blocks. The ELAN module incorporates four rounds of grouped convolution and employs skip connections in the internal residual structure, mitigating the issue of gradient vanishing often encountered in deep neural networks. The Downsampling-MP1 module is formed by concatenating two branches while maintaining an equal number of output channels as the input. Following the processing by the main network, we obtain three effective feature layers that will be utilized for subsequent network construction. The shapes of these three effective feature layers are (80, 80, 512), (40, 40, 1,024), and (20, 20, 1,024).

The Neck component is designed to enhance the fusion of effective feature layers obtained from the Backbone at four different scales. Initially, the effective feature layers acquired from Stage5 are processed through the SPPCSPC module, reducing the channel count from 1,024 to 512 while maintaining the same size. Subsequently, the effective feature layers from Stage3, Stage4, and those processed through the SPPCSPC module are fed into the Re-BiC module for multi-scale feature fusion, resulting in feature maps with dimensions of (40, 40, 512). These feature maps are then subjected to processing through the CCSMA module to enhance more salient feature expressions through channel-wise and spatial means. Following this, the feature layers processed by the ELAN module, along with the effective feature layers from Stage2 and Stage3, are input into the Re-BiC module for further multi-scale feature fusion, resulting in feature maps with dimensions of (80, 80, 256). They are then processed through the CCSMA module once more. Next, the entire network undergoes downsampling, achieved through the ELAM module and Downsampling-MP2 module, followed by feature addition. Compared to the original Concatenation operation in YOLOv7, using the ADD operation not only saves computational costs but also overlays semantic information extracted earlier, highlighting the correct classification ratio and preserving the correctly activated regions from the original image. Following processing by the Neck section, three enhanced feature layers are obtained, with dimensions of (80, 80, 128), (40, 40, 256), and (20, 20, 512), respectively.

Finally, these three enhanced feature layers are passed into the Head component, processed through RepConv layers, and then transmitted to the YOLOHead for generating prediction boxes. Given that the Su22kV_broken dataset used in this study only includes the Su22_Broken class, the final shapes of the three feature layers are (80, 80, 18), (40, 40, 18), and (20, 20, 18). And then, decoding is applied, followed by score sorting and non-maximum suppression to generate the optimal prediction boxes that meet the confidence threshold.

## 4 Experimental results

To assess the effectiveness of our proposed improved YOLOv7 method, we conduct model training and testing on both the Su22kV_broken dataset and the PASCAL VOC 2007 dataset. We also compare our approach with other mainstream object detection models. This chapter primarily focuses on the specifics of our experimental setup and methodology.

### 4.1 Datasets

**Su22kV_broken (Hieulc@cpc.vn, 2022)**. Section 3.1 provides comprehensive details regarding the Su22kV_broken dataset, comprising a total of 1,236 images, each with a resolution of 512 × 512.

**PASCAL VOC 2007 (Everingham et al., 2007)**. The PASCAL VOC 2007 dataset, an iteration of the PASCAL Visual Object Classes Challenge competition, holds significant prominence in the field of computer vision. This dataset serves as a pivotal resource for training, evaluation, and benchmark testing in various computer vision tasks, including object detection, image segmentation, and scene classification. It encompasses a total of 9,963 images with diverse pixel dimensions, encompassing a wide spectrum of object categories, scenes, and complexities. Post-annotation, these images are partitioned into training, validation, and test sets, comprising 2,501, 2,510, and 4,952 images, respectively. There are annotations for a total of 20 common object classes, with each image accompanied by an XML-formatted annotation file that includes information about object bounding box coordinates and class labels.

### 4.2 Implementation details

#### 4.2.1 Experimental environment

All of our experiments conduct in the same environment. The hardware environment includes a CPU [12th Gen Intel(R) Xeon(R) Platinum 8255C 2.50 GHz] and a GPU (NVIDIA GeForce RTX 2080 Ti), and the deep learning framework PyTorch and Python are used in the software environment.

#### 4.2.2 Training and evaluation metric

##### 4.2.2.1 Training

During the model training process, we set the momentum to 0.9 and weight decay to 5e-4. We use the Stochastic Gradient Descent (SGD) algorithm as the optimizer. For both the Su22kV_broken dataset and the PASCAL VOC 2007 dataset, we set the batch size to 16, the number of epochs to 200, and the initial learning rate to 0.01. Additionally, in the Su22kV_broken dataset, since we only need to detect the defect areas of insulators as a single target class, we calculate both the classification loss and localization loss.

##### 4.2.2.2 Evaluation metric

In this paper, we employ the commonly used performance evaluation metric in the field of object detection, Mean Average Precision (mAP), to assess the effectiveness of our algorithm. Here, we provide a brief introduction to the relevant metrics involved in computing mAP: Intersection Over Union (IOU), Precision, Recall, and Average Precision (AP).

IOU is a metric used to evaluate the extent of overlap between two bounding boxes. It reflects the localization accuracy of the predicted bounding box in relation to the true labeled box. If we denote the predicted bounding box as *A* and the true labeled box as *B*, the calculation of the Intersection Over Union is shown in Equation (14):


(14) $$\begin{array}{l} IOU=\frac{A\cap B}{A\cup B} \end{array}$$

In the formula, the numerator represents the area of overlap between *A* and *B*, while the denominator represents the sum of their individual areas minus the area of overlap.

Precision and recall are calculated separately for each class in the object detection task. If a predicted bounding box has a maximum IOU with all true labels greater than a threshold, it is considered a correct prediction; otherwise, it is considered an incorrect prediction. Each predicted box is associated with a confidence score, which is used to classify them into positive samples and negative samples. Calculating the IOU between predicted results and true labels yields the following: True Positives (*TP*), False Positives (*FP*), True Negatives (*TN*) and False Negatives (*FN*). Then the precision rate and recall rate are calculated, respectively, as shown in Equations (15) and (16):


(15) $$\begin{array}{l} Precision=\frac{TP}{TP+FP} \end{array}$$

(16) $$\begin{array}{l} Recall=\frac{TP}{TP+FN} \end{array}$$

From the above equation, it can be seen that precision in object detection tasks represents the extent of algorithmic false detection. Higher precision implies fewer false detections. On the other hand, recall represents the extent of algorithmic omission in object detection tasks. Higher recall implies fewer omission. The performance of an object detection algorithm should be evaluated considering both precision and recall. By setting different confidence thresholds can get different precision rate and recall rate, and connect them to form a curve called PR curve (the vertical axis is the precision rate, and the horizontal axis is the recall rate), the area of the closed region formed by the PR curve and the axes is the average precision rate (AP), if the curve corresponds to the function is notated as *p*(*r*), then the AP formula is shown in Equation (17).


(17) $$AP=\int_{0}^{1}p(r)dr$$

The mean Average Precision (mAP) is the average of the computed Average Precision (AP) values for all target categories. In this paper, as we are only detecting one category, which is defective insulators, mAP is equivalent to AP. In the field of object detection, 0.5 is commonly used as IOU threshold, and the mAP at this threshold is denoted as mAP@0.5.

### 4.3 Ablation study

This paper introduces several improvements to the original YOLOv7 model, including Edge Detail Shape Data Augmentation, CCSMA, Reconstructed Neck and the MPDIoU loss function. To individually assess the effectiveness of each of these improvements, we conduct ablation experiments on the test set of the Su22kV_broken dataset.

**Figure 8:**
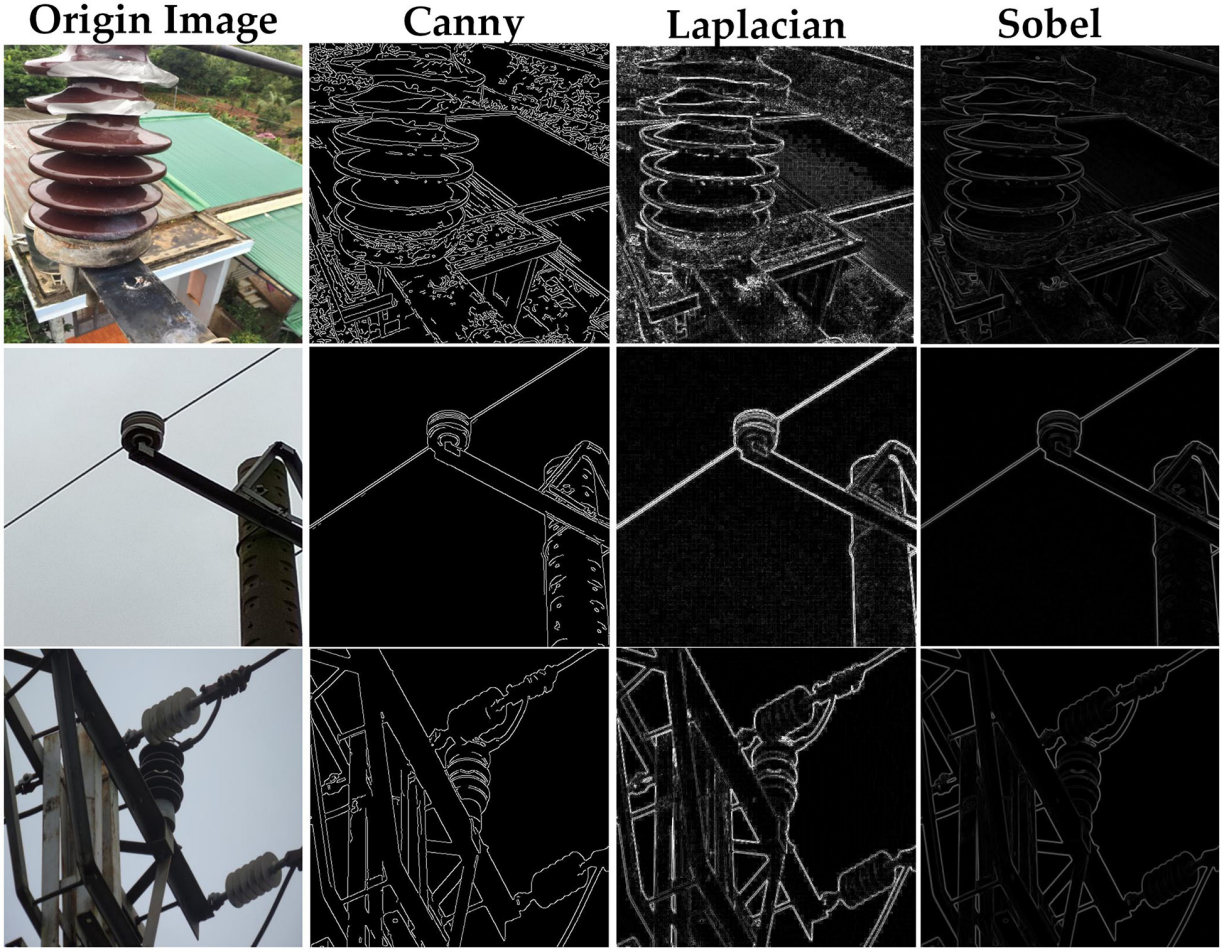
Results of edge details extracted by different algorithms.

In the experiments to validate the Edge Detail Shape Data Augmentation (EDSDA) module, we select and visualize three classic edge detection algorithms, as shown in Figure 8. These algorithms include the Canny operator (Ding and Goshtasby, 2001), the Laplacian operator (Wang, 2007), and the Sobel operator (Kanopoulos et al., 1988). The Canny operator can accurately extract fine edges, but it comes with a relatively high computational cost and requires parameter tuning for optimal results. The Laplacian operator, as a second-order differential operator, is adaptable to various edge scenarios without being restricted by edge direction, but it is sensitive to noise and can be affected by image noise. On the other hand, the Sobel operator offers faster computational speed, making it suitable for real-time applications. It has a certain degree of suppression effect on noise. Although it may not be as effective at detecting fine edges, but in general, it can capture the outer contours of insulators of different sizes and resolutions, and presents excellent detection performance.

Furthermore, to validate the effectiveness of the edge detail shape data augmentation method, we compare the performance of the original YOLOv7 model with the YOLOv7 model that incorporates the edge detail shape data augmentation module on the insulator dataset, as shown in Table 1. The experimental results demonstrate a significant improvement in the detection performance of the YOLOv7 model when the edge detail shape data augmentation module is employed. Among the methods tested, utilizing the Sobel operator achieved the highest mAP value, reaching 83.2%. It is noteworthy that among the Canny operator, which exhibited the highest edge detection accuracy, demonstrated a relatively lower mAP value of only 81.9%. This is likely because insulator defect detection typically places more emphasis on the edge contours of insulators, while the edge detail images generated by the Canny operator encompass a substantial amount of fine details and texture information beyond the insulators. Especially in the presence of complex backgrounds, it may introduce numerous non-insulator edges, thereby impacting the accuracy of detection. On the other hand, the Laplacian operator is susceptible to noise and may introduce false edge structures when generating edge images, consequently diminishing the reliability and accuracy of insulator defect detection.

**Table 1:** Ablation comparative experiment of edge detail shape data augmentation.

**Method**	**EDSDA**			**mAP@0.5(%)**
	**Sobel**	**Canny**	**Laplacian**	**Su22kV_broken**
YOLOv7	✓	**-**	**-**	**83.2**
	**-**	✓	**-**	81.9
	**-**	**-**	✓	82.6

Bold values indicate the best results from experiments with different algorithms.

Next, we conducte a series of ablation experiments on the proposed Reconstructed Neck component (Re-Neck, including Re-BiC module), CCSMA module, and the inclusion of the MPDIoU loss function during model training, as presented in Table 2. Building upon the original YOLOv7 framework, the implementation of the Re-Neck yields an mAP of 79.3%, indicating an improvement of 0.8% compared to the baseline YOLOv7. Subsequently, after introducing the CCSMA module and MPDIoU loss function on the basis of the Re-Neck component, respectively, the mAP values are 80.8 and 80.2%, which are improved by 1.5 and 0.9% respectively. With the simultaneous integration of all four methods, the mAP reaches 85.7%, showcasing a substantial 7.2% enhancement compared to the baseline YOLOv7. It is worth noting that using only the EDSDA (Sobel) data enhancement method on the basis of the Re-Neck component resulted in a 4.5% increase in mAP. This demonstrates that data with added edge information substantially improves detection in insulator defect detection tasks, thus proving the effectiveness of our data enhancement approach. The results of the ablation experiments affirm the satisfactory performance achieved by our proposed approach.

**Table 2:** Ablation experiments for the ID-YOLOv7 method.

**Method**	**Re-neck**	**EDSDA**	**CCSMA**	**MPDIoU**	**mAP@0.5(%)**
YOLOv7	**-**	**-**	**-**	**-**	78.5
ID-YOLOv7	✓	**-**	**-**	**-**	79.3
	✓	✓	**-**	**-**	83.8
	✓	**-**	✓	**-**	80.8
	✓	**-**	**-**	✓	80.2
	✓	✓	✓	**-**	85.2
	✓	**-**	✓	✓	81.5
	✓	✓	✓	✓	**85.7**

Bold values indicate the best results from experiments with different algorithms.

#### 4.3.1 Compare with state-of-arts on Su22kV_broken

We test our trained model on the Su22kV_broken dataset, and Table 3 presents the results of various evaluation metrics for the ID-YOLOv7 method compared to other mainstream object detection models on the test set. From the data in the table, it is evident that our proposed ID-YOLOv7 method achieved the best experimental results on the Su22kV_broken test set, with an mAP of 85.7%, which is 7.2% higher than Original YOLOv7. This demonstrates the significant advantages of our method in the task of insulator defect detection.

**Table 3:** Comparison results of different models.

**Method**	**Backbone**	**Precision (%)**	**Recall (%)**	**mAP@0.5 (%)**
Faster RCNN (Ren et al., 2015)	ResNet-50	82.5	69.6	71.4
SSD (Miao et al., 2019)	VGG-16	79.3	62.7	68.6
YOLOv5s (Jocher, 2020)	CSPDarknet	83.7	68.5	72.9
YOLOv7 (Wang C.-Y. et al., 2023)	-	87.5	73.1	78.5
YOLOv8 (Jocher et al., 2023)	CSPDarknet	85.7	73.8	77.8
Ours	-	**92.6**	**80.1**	**85.7**

Bold values indicate the best results from experiments with different algorithms.

**Figure 9:**
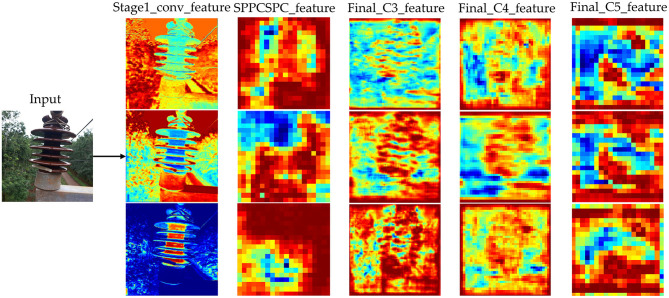
Comparison of training loss values for different models.

Next, we visualize the output feature maps of each layer in the ID-YOLOv7 model, as depicted in Figure 9. We present the feature maps from the network's first convolutional layer, SPPCSPC layer, and three feature-enhancing layers. From the images, it is evident that the features extracted by the first convolutional layer of the network exhibit a pronounced focus on the contours of the insulator edges, underscoring the effectiveness of our proposed edge detail shape data augmentation approach. As the network's depth increases, image features become increasingly dispersed. However, it is also evident from the feature maps of the three effective feature-enhancing layers that they exhibit excellent suppression of non-insulator features.

**Figure 10:**
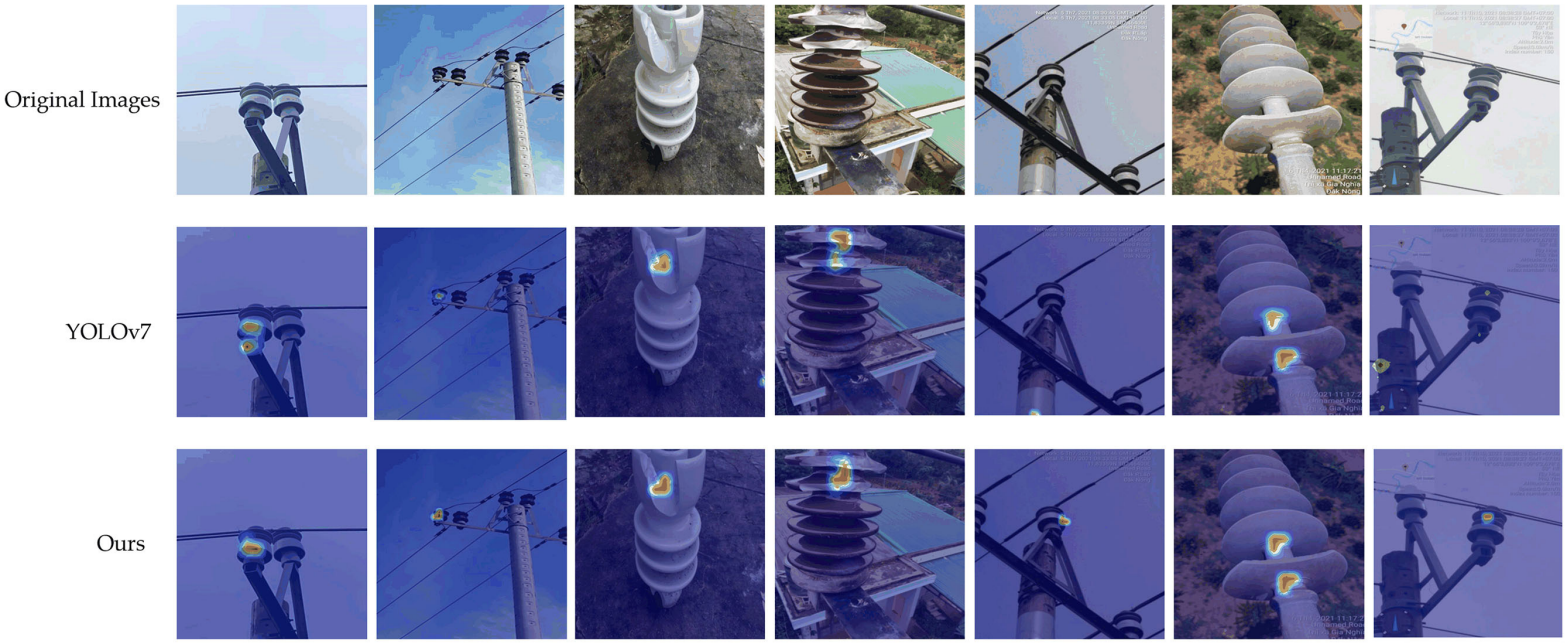
Visualization of feature map.

In addition, we employ the Grad-Cam (Selvaraju et al., 2017) algorithm to generate Grad-Cam maps for the original YOLOv7 model and the ID-YOLOv7 model, as illustrated in Figure 10. Notably, from these images, it becomes evident that our ID-YOLOv7 model is more adept at focusing on the subtle features of insulator defects within complex backgrounds. This suggests that the ID-YOLOv7 model holds a distinct advantage in insulator defect detection tasks.

**Figure 11:**
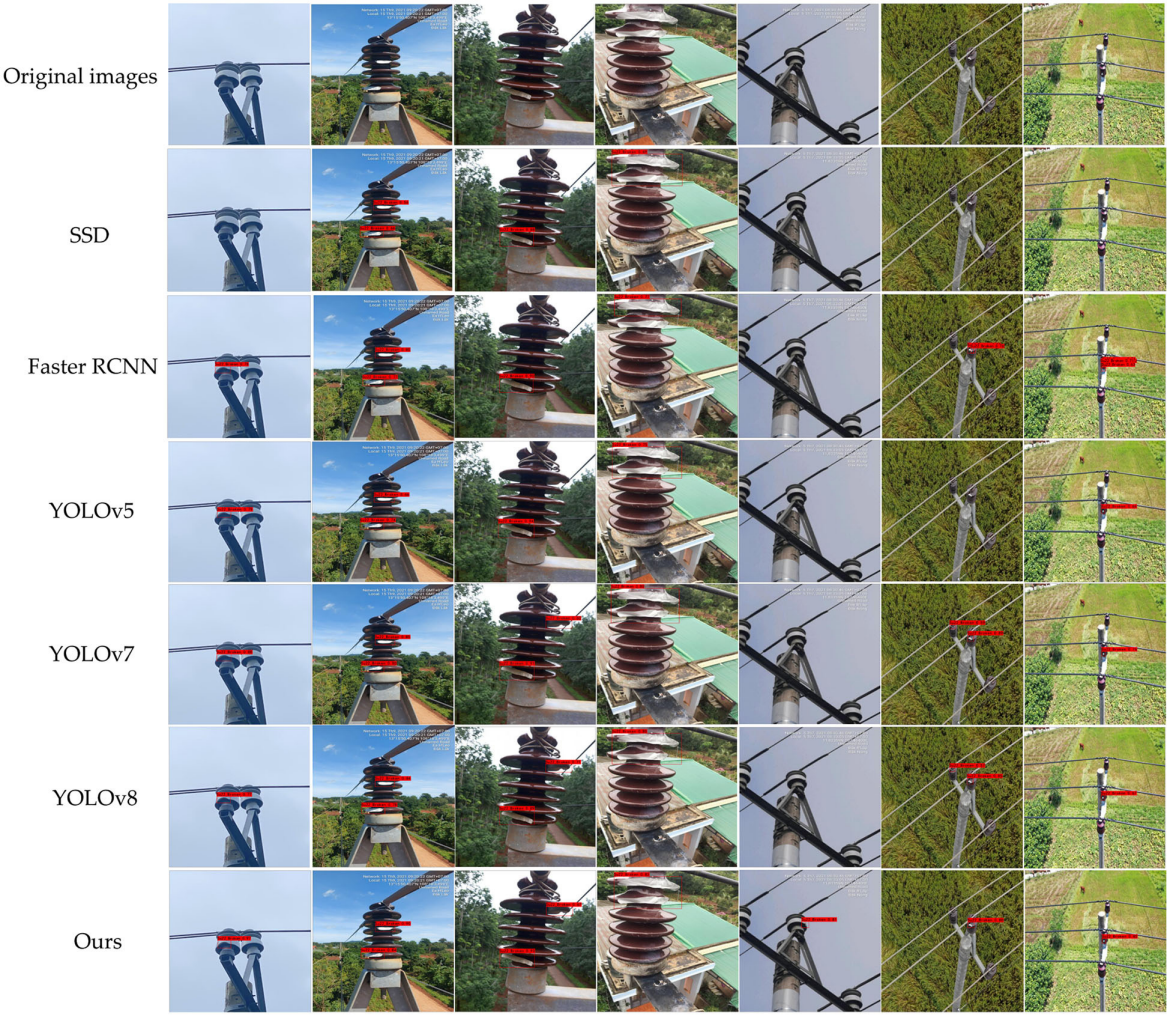
Grad-Cam map of YOLOv7 and our proposed ID-YOLOv7 model.

To further validate the sophistication of our proposed method, we present the detection results of different models for insulator defects, as shown in Figure 11. It is evident that SSD, Faster R-CNN, YOLOv5s, YOLOv7 and YOLOv8 models all exhibit varying degrees of omissions and false detections. Specifically, SSD demonstrates the poorest detection performance, Faster R-CNN exhibits redundant bounding boxes, and generally, the predicted bounding boxes have low confidence scores. Moreover, when it comes to detecting subtle defects, YOLOv5, YOLOv7, and YOLOv8 all exhibit instances of omissions and false detections. In contrast, our method not only addresses the issues of false negatives and false positives in insulator defect detection but also achieves high precision in accurate predictions. Therefore, it can be concluded that our proposed ID-YOLOv7 method is highly effective in insulator defect detection tasks.

#### 4.3.2 Compare with the mainstream methods on PASCAL VOC 2007

To further evaluate the effectiveness of our proposed ID-YOLOv7 model in object detection tasks, we conduct training on the PASCAL VOC 2007 dataset and compare the results on the test set with mainstream object detection algorithms. As shown in Table 4, our method achieves the highest mAP value on the PASCAL VOC 2007 dataset, reaching 90.3%, which is 2.9% higher than the original YOLOv7. Furthermore, the FPS also reaches 53, meeting the requirements of most real-world detection tasks.

**Table 4:** Comparison of different models on the PASCAL VOC 2007 dataset.

**Method**	**Backbone**	**mAP@0.5 (%)**	**FPS**
Fast RCNN (Girshick, 2015)	VGG-16	70.0	7
Faster RCNN (Ren et al., 2015)	ResNet-101	76.4	5
YOLOv2 (Redmon and Farhadi, 2017)	Darknet-19	78.6	40
SSD500 (Miao et al., 2019)	VGG-16	77.2	46
YOLOv3 (Redmon and Farhadi, 2018)	Darknet-19	64.8	37
YOLOv4 (Bochkovskiy et al., 2020)	Darknet-53	78.6	35
YOLOv5s (Jocher, 2020)	CSPDarknet	79.2	36
YOLOv7 (Wang C.-Y. et al., 2023)	-	87.4	51
Ours	-	**90.3**	**53**

Bold values indicate the best results from experiments with different algorithms.

## 5 Conclusion

In this article, we address the challenges posed by the complex backgrounds and numerous subtle defects in insulator images captured by drones in power distribution network. Building upon the YOLOv7 algorithm, we propose an improved version for the detection of insulator defects. Extensive experiments and visual results substantiate the effectiveness of our approach. On the Su22kV_broken dataset, we achieve mAP of 85.7% using a single NVIDIA RTX 2080ti graphics card, which is 7.2% higher than the original YOLOv7. On the PASCAL VOC 2007 dataset, we achieve a remarkable mAP of 90.3% at a speed of 53 FPS. In comparison to other mainstream object detection algorithms, our method demonstrates significant advantages.

In future work, we intend to further improve our dataset by incorporating diverse insulator defect data from various environmental conditions. Additionally, we will continue our research to develop high-precision insulator defect detection algorithms that meet real-time performance requirements, with the ultimate goal of contributing to the stable and safe operation of power systems.

## Data availability statement

The datasets presented in this study can be found in online repositories. The names of the repository/repositories and accession number(s) can be found below: https://universe.roboflow.com/hieulc-cpc-vn/su22kv_broken; http://host.robots.ox.ac.uk/pascal/VOC/voc2007/index.html.

## Author contributions

BC: Conceptualization, Methodology, Writing – original draft. WZ: Methodology, Software, Writing – original draft. WW: Validation, Writing – review & editing. YL: Funding acquisition, Writing – review & editing. ZC: Visualization, Writing – review & editing. CL: Conceptualization, Writing – review & editing.
